# Parathyroid hormone-related protein serves as a prognostic indicator in oral squamous cell carcinoma

**DOI:** 10.1186/s13046-014-0100-y

**Published:** 2014-12-18

**Authors:** Zhongjing Lv, Xiangbing Wu, Wei Cao, ZongZe Shen, Lizhen Wang, FuRong Xie, JianJun Zhang, Tong Ji, Ming Yan, WanTao Chen

**Affiliations:** Department of Oral and Maxillofacial-Head and Neck Oncology, Ninth People’s Hospital, Shanghai Jiao Tong University School of Medicine, Shanghai, China; Shanghai Key Laboratory of Stomatology and Shanghai Research Institute of Stomatology, Shanghai, China; Department of Oral Pathology, Ninth People’s Hospital, Shanghai Jiao Tong University School of Medicine, Shanghai, China

**Keywords:** PTHrP, PTHLH, OSCC, Cell cycle, Biomarker

## Abstract

**Background:**

In our previous study, parathyroid hormone-like hormone (PTHLH) which encodes parathyroid hormone-related protein (PTHrP) was revealed to be up-regulated in oral squamous cell carcinoma (OSCC) compared with paired apparently normal surgical margins using microarray method. However, the function and prognostic indicators of PTHLH/PTHrP in OSCC remain obscure.

**Methods:**

The mRNA levels of PTHLH and its protein levels were investigated in 9 OSCC cell lines and in 36 paired OSCC specimens by real-time PCR and western blotting. The biological function of PTHLH/PTHrP was investigated using small interfering RNA (siRNA) in 3 OSCC cell lines, and immunohistochemistry was used to estimate the prognostic value of PTHrP in 101 patients with head and neck squamous cell carcinoma (HNSCC), including OSCC and oropharyngeal squamous cell carcinoma. Cell cycle was tested by flow cytometry and cell cycle related genes were investigated by western blotting and immunocytochemistry assay.

**Results:**

This study showed that the mRNA and protein levels of PTHLH in 9 OSCC cell lines were much higher than that in normal epithelial cells (*P* < 0.0001). In 36 paired OSCC tissues, PTHLH mRNA expressions were found higher in 32 OSCC tissues than that of paired apparently normal surgical margins (*P* = 0.0001). The results revealed that the down-regulation of PTHLH/PTHrP by siRNAs could reduce cell proliferation and inhibit plate and soft agar colony formation as well as affect the cell cycle of OSCC cells. The key proteins related to the cell cycle were changed by anti-PTHLH siRNA. The results showed that cyclin D1 and CDK4 expressions were significantly reduced in the cells transfected with anti-PTHLH siRNA. On the other hand, the expression of p21 was increased. The results also showed that high PTHrP level was associated with poor pathologic differentiation (*P* = 0.0001) and poor prognosis (*P* = 0.0003) in patients with HNSCC.

**Conclusions:**

This study suggests that PTHLH/PTHrP is up-regulated in OSCCs. Therefore, PTHLH/PTHrP could play a role in the pathogenesis of OSCC by affecting cell proliferation and cell cycle, and the protein levels of PTHrP might serve as a prognostic indicator for evaluating patients with HNSCCs.

## Background

Head and neck cancer (HNC), the sixth-most common cancer in the world, seriously harm to the human health. The global incidence of HNC has increased recently, according to the report; more than 500,000 individuals were involved each year [1,2]. Oral cancer is the most common HNC and oral squamous cell carcinoma (OSCC) account for >80% of HNC. Along with a further understanding of the pathogenesis of OSCC and improved treatment, the survival rates of OSCC have improved over the past ten years; however, about 50% of patients still died of this disease within five years after diagnosis [3]. OSCC is often seriously threatened to human health because of its fast progression and the particular disease site. As is known, similar to other tumors, the pathogenesis of OSCC is a complicated process. Many factors, such as physical factors, chemical factors, immunity of the individual and so on, are involved in the pathogenesis of OSCC. With the development of modern molecular biological techniques, many genes, such as the oncogene *C-myc* and tumor suppressor gene p53, have been found to be abnormally expressed in OSCC [4,5]. Moreover, in our previous studies, we found that the MAL gene and TGM3 gene were down-regulated in OSCC [6,7]. And it has been reported that matrix metalloproteinases (MMPs) expression in oral tongue cell carcinoma were much higher than those in normal oral mucosa [8]. Recently, it has also been revealed that many cytokines and hormones participate in the development of cancer [9,10]. We can preliminary estimate the prognosis of OSCC based on the expression of these genes, but so far, there is still no ideal indicator to predict the prognosis for patients with OSCC. By studying the function of tumor-related genes, we not only reveal their roles in OSCC development but also evaluate the powers to predict the prognosis of patients with OSCC.

In our previous study, parathyroid hormone-like hormone (PTHLH) was found to be up-regulated 2.5-fold in 22 pairs of OSCCs compared with the apparently normal surgical margins through screening and oligonucleotide microarray analysis [11], which suggests that PTHLH may play an important role in the development of OSCC. Unlike parathyroid hormone (PTH), which is secreted only by the parathyroid gland, PTHrP, the protein encoded by PTHLH, is expressed and secreted by a few normal tissues and many tumor cells in an autocrine or paracrine way. The circulating concentrations of PTHrP are low or undetectable in normal tissues [12]. The first eight amino acids of PTHrP and PTH are the same, that is to say, PTHrP is a protein with N-terminal homology to PTH. PTHrP has the ability to combine and activate the PTH/PTHrP receptor in bone and, consequently, is a potent stimulant of osteoclasts and osteoblasts [13]. The expression of PTHrP had been studied in human endometrial stromal cells, and PTHrP has been shown to stimulate the proliferation of endometrial stromal cells *in vitro* [14,15]. It also had been revealed that certain doses of PTHrP not only relaxed vascular/nonvascular smooth muscle and oscillated blood flow but also regulated trans-epithelial calcium transport in various tissues [16,17]. In a recent study, it was found that PTHrP was up-regulated in many tumors and was responsible for paraneoplastic syndromes such as hypercalcemia in malignancy [18]. According to the literatures, PTHrP produced by cancer cells plays an important role in the development of bone metastases and hypercalcemia in breast and lung cancers [19,20]. More recently, it was suggested and confirmed by several studies that PTHLH/PTHrP might contribute to the proinflammatory cytokine cascade, resulting in elevated serum cytokines such as interleukin-6 and tumor necrosis factor [21,22]. Thus, PTHrP imbalance is implicated in the pathogenesis of cancer. However, the local biological function of PTHLH/PTHrP has not been reported in OSCC, and it remains unclear whether PTHrP can be used as a valuable prognostic biomarker for patients with OSCC.

In this study, to verify the role of PTHLH/PTHrP in OSCCs, the expression of PTHLH mRNA and PTHrP protein was measured in 9 OSCC cell lines and 36 OSCC tissues compared to the expression in normal primary oral epithelial cells and paired apparently normal surgical margins, respectively, using real-time PCR and western blotting. The biological function of PTHLH/PTHrP in OSCC was observed by knocking down PTHLH/PTHrP expression in the WSU-HN6, HN13 and CAL-27 cell lines. Furthermore, immunohistochemistry was used to evaluate the correlation between the PTHrP expression level and prognosis as well as clinical parameters. The data initially suggested that PTHLH could play an oncogenic role in the pathogenesis of OSCC and PTHrP could serve as a valuable prognostic indicator for OSCC.

## Methods

### Cell culture

The OSCC cell lines WSU-HN4, HN6, HN12, HN13 and HN30 (obtained from the University of Maryland Dental School, USA) and CAL-27 (provided by the American Type Culture Collection, USA) that were used in this study were cultured in Dulbecco’s Modified Eagle medium (DMEM) with 10% fetal bovine serum (FBS) (GIBCO-BRL, USA), streptomycin (100 μg/ml) and penicillin (100 units/ml). Normal epithelial cells of gingival tissues which taken from the patients with impacted teeth extraction were cultured in KSF (keratinocyte serum-free medium; GIBCO-BRL) with 0.2 ng/ml rEGF (recombinant epidermal growth factor; Invitrogen, USA). The SCC-4, SCC-9 and SCC-25 cell lines (purchased from the American Type Culture Collection, USA) were cultured in DMEM/12 medium (GIBCO-BRL) supplemented with 10% FBS, streptomycin and penicillin (the concentrations were the same as above). All cell lines were cultured in a humidified, 5% CO_2_ incubator at 37°C [23].

### Patients and specimens

In this study, pairs of samples were obtained from 36 patients who were first diagnosed with OSCC and had not undergone any treatment before visiting our hospital between 2008 and 2009. Upon obtaining the cancerous tissues and apparently normal surgical margins, the samples were immediately placed in liquid nitrogen to prevent RNA degradation. The diagnosis of each sample was confirmed before the extraction of total RNA and proteins. The tissue microarray samples were obtained from paraffin-embedded tissue of 101 patients with head and neck squamous cell carcinoma (HNSCC) who had undergone surgical treatment when first diagnosed with OSCC or oropharyngeal squamous cell carcinoma (OPSCC) between 1989 and 1993. In this study, the sample macrodissection has not been performed. The patients involved in this study were required to be primary HNSCC and no distant metastases. And the patients who accepted any treatments such as radiotherapy, chemotherapy and/or medicinal treatment were ruled out. The detailed information on those 101 patients was obtained to analyze the correlation between the expression levels of PTHrP and clinical parameters. Of 101 patients with HNSCC, the rest 99 patients had complete clinical information excepting 2 patients without follow-up data. Samples were stained with hematoxylin and eosin to analyze the pathological differentiation and pathological diagnosis which were confirmed by two experienced pathologists. In this study, pathological differentiation and clinical stage were respectively determined according to World Health Organization Classification of Tumors and the TNM classification system of the International Union Against cancer (1988). The patients involved in this study signed a written informed consent, and the work was approved by the Medical Ethics Committee of the Ninth People’s Hospital, Shanghai Jiao Tong University School of Medicine.

### Immunohistochemistry and immunocytochemistry

The tissues were fixed with formalin and embedded in paraffin then cut into 4 μm sections. The tissue sections were then deparaffinized in xylene and rehydrated in graded ethanol. For antigen retrieval, the samples were heated with citrate buffer for 15 minutes. The samples were then incubated with rabbit polyclonal PTHrP antibody (1:150; Bioworld, USA) or rabbit polyclonal p16 antibody (1:200; Proteinteck Group, USA) at 4°C overnight followed by incubation with a biotinylated secondary antibody and staining with Diaminobenzidine (DAB) (Dako, Denmark). The staining index was quantitatively divided into scores of 0, 1, 2, and 3 according to the intensity of staining. In this study, the scores representing the percentage of positive epithelial cells were obtained and expressed as 0-100% and were analyzed by two experienced pathologists. The total scores were obtained by multiplying the staining index by the percentage of positive epithelial cells [24]. The expressions of PTHrP were divided into high and low levels based on a median score of 2.55 which was the cutoff value for determining the PTHrP low or high expression. Thus, a value <2.55 was considered low expression, and a value ≥2.55 was considered high expression. For immunocytochemistry, cells were fixed by 4% paraformaldehyde and incubated with 0.1% Triton X-100 for 10 minutes, then stained with primary antibody and anti-rabbit secondary antibody, followed by stained with chromogenic agent DAB.

### RNA extraction

Total RNA was isolated from cell lines and frozen tissue samples using TRIzol reagent (Invitrogen, USA) according to the manufacturer’s instructions. The concentration and purity of the total RNA were measured by optical density measurements at 260 nm and 280 nm. The RNA was then stored at -80°C or converted to cDNA using SuperScript II reverse transcriptase (Takara, Japan) [25].

### Real-time polymerase chain reaction analysis

The real-time PCR reactions were accomplished using the ABI 7300 real-time PCR system (ABI, USA) with the SYBR Premix E × Tap reaction kit (Takara, Japan). Real-time PCR amplification was performed in a volume of 20 μl that included 1 μl of cDNA template, 10 μl of SYBR Premix E × Tap, 0.4 μl of ROX Reference Dye, 0.4 μl each of the forward and reverse primers and 7.8 μl of sterile distilled water. The following primers were used to amplify human PTHLH: the forward primer was 5’-CGTCTCCACCTTGTTAGTTTCC-3’ and the reverse prime was 5’-GCAGAAATCCACACAGCTGA-3’; the following primers were used to amplify β-actin: the forward primer was 5’-CCTGGCACCCAGCACAAT-3’ and the reverse primer was 5’-GGGCCGGACTCGTCATACT-3’. The real-time PCR program was carried out according to the instructions of the SYBR Premix E × Tap reaction kit. The results were analyzed using the 2^-ΔΔCt^ method; ΔCt was the difference between the Ct values of the target gene and the Ct values of β-actin, and ΔΔCt was the difference between the ΔCt of the OSCC samples minus the ΔCt of the apparently normal surgical margins. The results then indicated the relative expression of PTHLH in the OSCC samples compared with that of the apparently normal surgical margins [26].

### Western blot analysis

The cells were washed two times with cold phosphate-buffered saline (PBS) before harvesting and then lysed in sodium dodecyl sulfate (SDS) lysis buffer (Beyotime, China) containing complete protease inhibitors (Roche Bioscience, USA). Once the cells were completely lysed, the proteins were boiled, and their concentrations were analyzed using the Pierce BCA Protein Assay kit (Thermo Scientific, USA). Similar quantities of total proteins were electrophoresed by sodium dodecyl sulfate-polyacrylamide gel electrophoresis (SDS-PAGE) and then transferred to a 0.22-μΜ polyvinylidene fluoride (PVDF) membrane, which was blocked with non-fat milk for 1 hour at room temperature followed by incubation with primary antibody for 2 hours at room temperature. The membrane was then washed 3 times with PBS supplemented with 0.1% Tween-20 for 10 minutes each, and anti-mouse/rabbit secondary antibodies were used to detect specific immunoreactivity [27]. In this study, the rabbit polyclonal antibody against PTHrP was used (Bioworld, USA); and the rabbit polyclonal Cyclin D1 (CCND1) antibody (1:5000; Clone EPR2241, Epitomics Inc., USA), the rabbit polyclonal p21 antibody and the rabbit polyclonal CDK4 antibody were used (1:1000; Proteintech Group, USA). The mouse monoclonal β-actin (1:5000; Sigma-Aldrich, USA) served as a loading control.

### Small interfering RNA transfection

Small interfering RNAs (siRNA) specific for PTHLH were designed and chemically synthesized by Biomics Biotechnologies Co., Ltd. In this study, the sequence for the human PTHLH siRNA (HS-PTHLH-si; abbreviated as P-si) was 5’-GACGAUUCUUCCUUCACCAdTdT-3’. The scrambled siRNA was also obtained from Biomics Biotechnologies Co., Ltd. The OSCC cell lines were cultured in 6-well cell culture plates and transfected using Lipofectamine 2000 (Invitrogen, CarIsbad, USA) according to the manufacturer’s protocol.

### Cell proliferation assay

The OSCC cell lines that were transfected with the PTHLH siRNAs were transferred to triplicate wells of a 96-well cell culture plate at a density of 1 × 10^3^ cells/well. To analyze the differences in the optical densities (ODs) of the cell lines transfected with P-si or scrambled siRNA, a CCK-8 kit was used (Dojindo, Japan). The OD value was read at 450 nm 2 hours after CCK-8 was added following the kit instructions, and the cell viability was measured at intervals of 24 hours [28].

### Cell cycle analysis

The cells were cultured overnight in DMEM without serum after transfection with the siRNAs for 24 hours, replaced with DMEM containing 10% FBS for 24 hours and then harvested. The cells were then washed twice with cold PBS and fixed in cold 70% ethanol overnight according to the instructions of BD Company. The fixed cells were gently washed twice with cold PBS and stained with PI staining buffer for 30 minutes at 4°C in the dark. Finally, the cell cycle was analyzed by flow cytometry [24].

### Plate colony formation assay

siRNA-transfected cells (1 × 10^3^) were cultured with DMEM supplemented with 10% FBS in 10 cm culture dishes for two weeks. The cell colonies were washed twice with PBS and fixed with paraformaldehyde for 30 minutes. Then, the cells were stained with 0.1% Coomassie Brilliant Blue R-250 (Thermo scientific, USA) for more than 2 hours [29]. The colony formation assay was performed according to the size and density of the colonies to evaluate the proliferation of cells transfected with the siRNAs. In this study, 3 independent experiments were represented the colony formation ability.

### Soft agar colony formation assay

Twenty-four hours after cells transfected with siRNA, 3 × 10^3^ siRNA transfected cells were mixed with 0.5 ml of 0.5% agarose in DMEM with 10% FBS, then seeded on the top of solidified 1% agarose in 24-well plate in triplicates. The gel was covered with 200 μl of DMEM medium after the top layer was solidified, added 50 μl DMED medium every week to prevent the gel drying. The gels incubated for 3-4 weeks until the colonies formed obviously, then colonies >0.1 mm in diameter were counter under a microscopic field at × 20 magnification. 3 independent experiments were finished in this study.

#### *In situ* hybridization (ISH)

Human papilloma virus (HPV) 16 and 18 was detected by *in situ* hybridization according to the instruction of Human papilloma virus (16/18) detection kit (Triplex International Bioscience, China). Briefly, 4 μm tissue sections were deparaffinized in xylene and rehydrated in graded ethanol. Then, the samples were heated with hybrid enhanced buffer for 30 minutes, the slides were hybridized with a biotinylated HPV16/18-type-specific probe and degenerated for 5 minutes at 95°C, followed by being incubated for 16 hours at 37°C *in situ* hybridization heater. Then incubated in order with reagent A, B and C (Triplex International Bioscience, China) at 37°C and stained with chromogenic reagent DAB (Triplex International Bioscience, China).

### Statistical analyses

In this study, the results of the real-time PCR, cell proliferation assays, cell cycle assays, colony formation assays were compared using student’s *t*-test. One-way analysis of variance (ANOVA) was used to compare three groups. Kaplan-Meier analysis was used to estimate the PTHrP expression and overall survival. The Cox proportional hazards model was used for univariate and multivariate analyses of the prognosis of patients. In this study, all computation was accomplished using SPSS software 11.0 and GraphPad Prism, and all statistical tests were two-sided. *P* < 0.05 was considered statistically significant.

## Results

### PTHLH/PTHrP was up-regulated in OSCC specimens and cell lines

**Figure 1:**
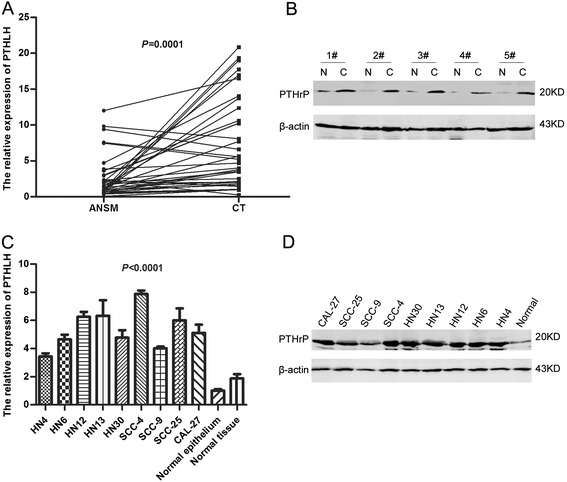
**PTHLH mRNA and PTHrP protein expression in OSCC specimens and cell lines. (A)** PTHLH mRNA expression was analyzed in 36 paired OSCC specimens including cancerous tissues and apparently normal surgical margins using real-time PCR (Mean ± SE: 2.618 ± 0.495, rang: 0.0817 ± 12.010 vs. Mean ± SE: 7.376 ± 1.040, rang: 0.988 ± 20.873; *P* = 0.0001; two-tailed Student’s *t*-test). **(B)** PTHrP protein expression was determined in 5 representative paired OSCC specimens by western blotting analysis (N, apparently normal surgical margin; C, cancerous tissue). **(C)** PTHLH mRNA levels were determined in 9 representative OSCC cell lines, primary normal epithelial cells and normal tissues showing in Figure 1A using real-time PCR (*P* < 0.0001; One-way ANOVA). **(D)** PTHrP protein levels were determined in 9 representative OSCC cell lines and primary normal epithelial cells using western blotting.

To determine if PTHLH/PTHrP was up-regulated in OSCC, the PTHLH mRNA levels were investigated in 36 paired OSCC specimens using real-time PCR. The results showed that PTHLH was up-regulated in 88.9% (32/36) of OSCC tissues compared with the apparently normal surgical margins (*P* = 0.0001) (Figure 1A). Then, western blot analysis was used to analyze the PTHrP protein levels in 5 representative paired OSCC specimens and further confirmed that PTHrP was up-regulated in OSCC specimens (Figure 1B). Moreover, we analyzed the PTHLH mRNA and PTHrP protein levels in OSCC cell lines and primary normal epithelial cells. These results revealed that the PTHLH mRNA and PTHrP protein levels were up-regulated in all 9 OSCC cell lines compared with the normal epithelial cells (*P* < 0.0001) (Figure 1C, D).

### Down-regulation of PTHLH/PTHrP could inhibit cell proliferation and colony formation

**Figure 2:**
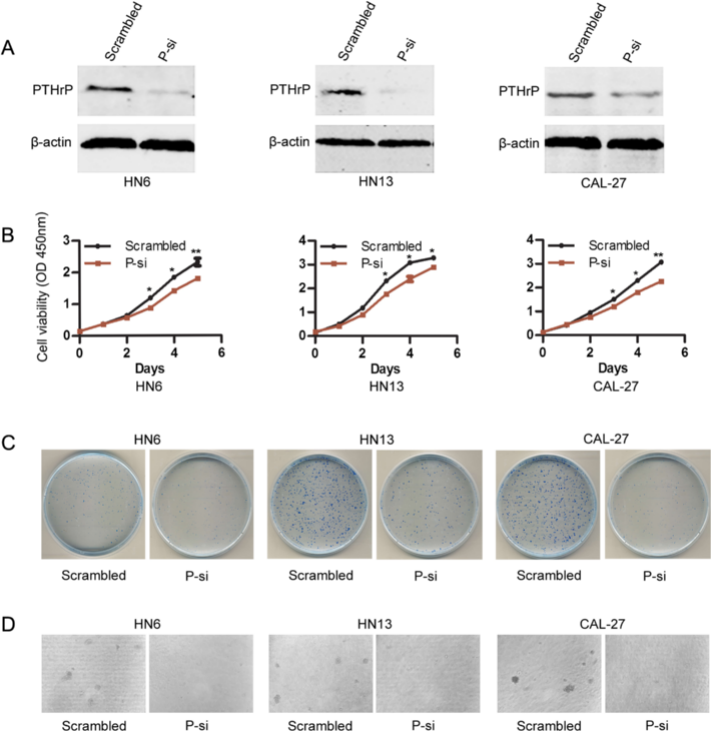
**The effect of PHTLH/PTHrP on cell proliferation and colony formation. (A)** PTHrP protein expression was confirmed in WSU-HN6, HN13 and CAL-27 cell lines transfected with anti-PTHLH siRNA P-si or scrambled siRNA using western blotting. **(B)** Cell proliferation of WSU-HN6, HN13 and CAL-27 cell lines transfected with anti-PTHLH siRNA P-si or scrambled siRNA were analyzed using the CCK-8 kit, each point showed the mean values of triplicate wells (Mean ± SE). **(C)** Plate colony formation of WSU-HN6, HN13 and CAL-27 cell lines transfected with anti-PTHLH siRNA P-si or scrambled siRNA was analyzed. **(D)** Soft agar colony formation assay was analyzed between the cell lines transfected with anti-PTHLH P-si and cell lines transfected with scrambled siRNA.

Based on the results showing that PTHLH/PTHrP was up-regulated in all 9 OSCC cell lines, siRNAs were used to analyze the biological function of PTHLH/PTHrP in OSCC cell lines *in vitro*. Three cell lines, WSU-HN6, HN13 and CAL-27, were transiently transfected with siRNAs (Figure 2A), and the results showed that the growth of cells transfected with P-si was obviously reduced compared to that of cells transfected with scrambled siRNA (Figure 2B). This data implied that the overexpression of PTHLH/PTHrP in OSCC cell lines promoted cell proliferation. Considering PTHLH/PTHrP could affect cell proliferation *in vitro*, plate colony formation assays and soft agar colony formation assays were performed to further confirm these results. The efficiency of plate colony formation results indicated that there was a significant difference between the cell lines transfected with P-si and those transfected with the scrambled siRNA, the colony number of the former was obviously less than that of the latter (Figure 2C). Soft agar colony formation assay also showed that colonies of cells transfected with P-si were much less and smaller than that of cells transfected with scrambled siRNA (Figure 2D). The results, to some extent, further confirmed that down-regulation of PTHLH/PTHrP could reduce the cell proliferation of OSCC cell lines.

### Down-regulation of PTHLH/PTHrP blocked the cell cycle of OSCC cell lines

**Figure 3:**
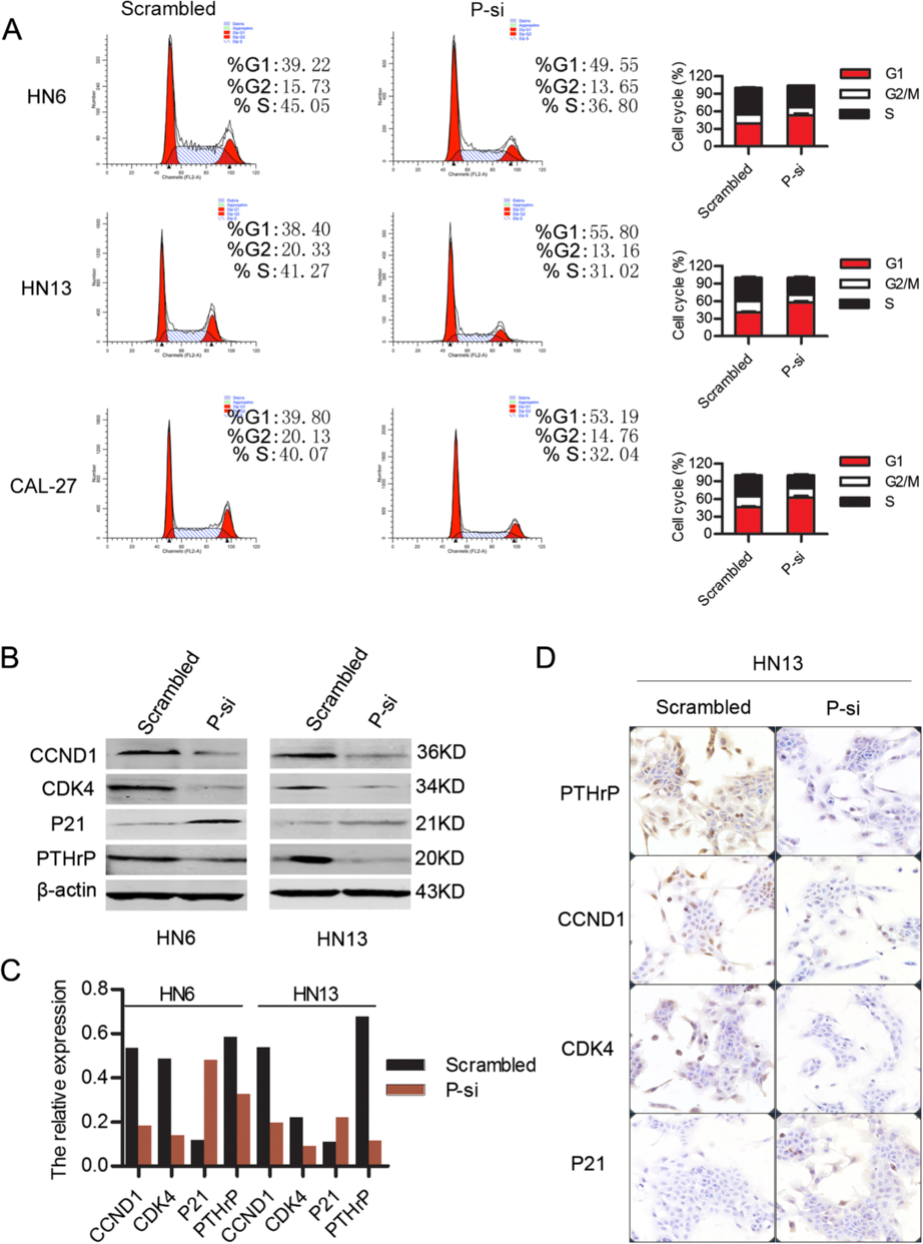
**The impact of PTHLH/PTHrP on the cell cycle. (A)** Cell cycle was analyzed in WSU-HN6, HN13 and CAL-27 cell lines transfected with anti-PTHLH siRNA P-si or scrambled siRNA. **(B)** The proteins of Cyclin D1, CDK4 and p21 which related to cell cycle were determined in WSU-HN6 and HN13 cell lines transfected with anti-PTHLH siRNA P-si or scrambled siRNA. **(C)** The fold change in western blot (Figure B) was quantified and analyzed. **(D)** Immunocytochemistry staining of cells transfected with PTHLH-siRNA was used to analyze the expression of PTHLH, Cyclin D1, CDK4 and p21.

To explore the underlying mechanism for the reduction of cell proliferation and colony formation due to down-regulation of PTHLH/PTHrP, the cell cycles of WSU-HN6, HN13 and CAL-27 cells transfected with P-si or scrambled siRNA were analyzed and assessed by flow cytometry. The results showed that the fractions of P-si transfected WSU-HN6, HN13 and CAL-27 cells in G1 phase were increased to 49.55%, 55.80% and 53.19%, respectively compared to the corresponding cells transfected with scrambled siRNA (39.22%, 38.40% and 39.80%, respectively) (*P* < 0.05) (Figure 3A). This result suggested that down-regulation of PTHLH/PTHrP reduced the cell proliferation index of the OSCC cell lines.

To further confirm that PTHLH/PTHrP affects the cell cycle of OSCC cell lines, key proteins related to the cell cycle in cells transfected with P-si or scrambled siRNA were measured by western blotting and immunocytochemistry. The results showed that Cyclin D1 (CCND1) and CDK4 were significantly reduced in the cell lines transfected with P-si. On the other hand, the expression of p21 was increased (Figure 3B, C, D). These results indicated that down-regulation of PTHLH/PTHrP inhibited cell proliferation and colony formation by blocking the cell cycle of OSCC cells at G1 phase *in vitro*. These preliminary results indicated that the overexpression of PTHLH/PTHrP played a key role in the pathogenesis of OSCC.

### The overexpression of PTHrP was associated with poor pathological differentiation and poor overall survival in patients with HNSCC

**Figure 4:**
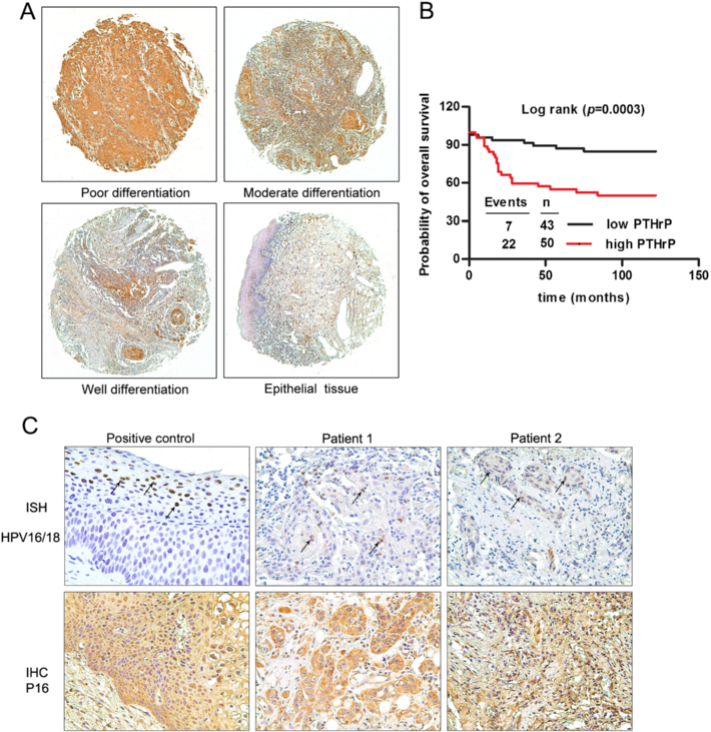
**The clinical significance of PTHrP overexpression was analyzed in 93 patients with HNSCCs. (A)** PTHrP expression was detected in poorly differentiated, moderately differentiated and well differentiated specimens and in normal epithelial specimens, as shown by immunohistochemistry. **(B)** Kaplan-Meier survival curves illustrating the overall survival of 93 patients with HNSCC according to the PTHrP protein levels. **(C)** Two patients with base of tongue squamous cell carcinoma in this study were positive for HPV16/18 and p16 expression using ISH and IHC methods.

To further analyze the clinical significance of the overexpression of PTHLH/PTHrP in patients with HNSCC, the expression of PTHrP was measured using a tissue microarray containing 101 HNSCC specimens and 10 normal tissues by means of immunohistochemistry. Excepting for 6 patients were taken off the pieces in the process of IHC and 2 patients had no follow-up data, 64 patients survived and 29 patients died in the rest of 93 patients. The follow-up time was from 1 month to 122 months with the median follow-up time of 84 months. PTHrP proteins were expressed in the cytoplasm to different degrees in HNSCC specimens. The expression of PTHrP in HNSCC tissues was much higher than in normal epithelial tissues. Moreover, the highest PTHrP protein staining of all specimens was observed in poorly differentiated HNSCCs. The results showed that the PTHrP expression level was positively correlated with pathological differentiation: the poorer the pathological differentiation, the stronger the PTHrP protein staining in the 93 HNSCC specimens (*P* = 0.0001) (Figure 4A). The analysis showed no significant differences between the PTHrP expression and age, gender, TNM classification, lymph node metastasis and the site of the disease (Table 1). The overall survival probability of the 93 HNSCC patients was analyzed using Kaplan-Meier and Cox proportional hazards regression models. The results showed that patients with highly expressing PTHrP had poorer survival than those with low PTHrP expression (*p* = 0.0003) (Figure 4B). The univariate and multivariate Cox proportional hazards regression models showed that not only pathological differentiation but also the PTHrP expression level could serve as an independent indicator of prognosis for patients with HNSCC (Table 2).

**Table 1:** **Demographic characteristics of the patient population by PTHrP expression**

**Characteristic**	**N (%)**	**PTHrP expression (M ± SD)**	***P***
**Age**			
<60y	54 (58%)	2.757 ± 0.391	0.6775^a^
≥60y	39 (42%)	2.723 ± 0.391	
**Gender**			
Male	49 (52.7%)	2.353 ± 0.459	0.5592^a^
Female	44 (47.3%)	2.283 ± 0.633	
**Smoking history**			
No	62 (66.7%)	2.706 ± 0.460	0.5850^a^
Yes	31 (33.3%)	2.754 ± 0.352	
**Alcohol history**			
No	74 (79.6%)	2.755 ± 0.382	0.4071^a^
Yes	19 (20.4%)	2.671 ± 0.436	
**TNM stage**			
I/II	48 (51.6%)	2.380 ± 0.558	0.7570^a^
III/IV	45 (48.4%)	2.346 ± 0.515	
**Disease site**			
Tongue	64 (68.8%)	2.348 ± 0.540	0.4972^b^
Base of tongue	7(7.5%)	2.407 ± 0.480	
cheek	11(11.8%)	2.231 ± 0.514	
gingiva	9(9.7%)	2.478 ± 0.607	
Orthers (lip, floor of mouth)	2 (2.2%)	2.732 ± 0.458	
**Lymph node metastasis**			
pN0	64 (68.8%)	2.740 ± 0.392	0.8158^a^
pN1-pN2	29 (31.2%)	2.761 ± 0.393	
**Pathological differentiation**			
I	54 (58.1%)	2.189 ± 0.540	0.0001^a^
II/III	39 (41.9%)	2.610 ± 0.434	

^a^These *P* value were analyzed by two-tailed Student’s *t*-test.

^b^These *P* value were analyzed by One-way ANOVA analysis.

**Table 2:** **Univariate and multivariate Cox Regression Models for estimating the overall survival**

**Characteristics**	**HR**	**95% CI**	***P***
**Univariate analysis**			
Overall survival			
Age (<60 y vs ≥60 y)	0.558	0.254 to 1.225	1.146
Gender (male vs female)	0.935	0.880 to 4.252	0.100
Alcohol history (nonsmoker vs smoker)	1.543	0.683 to 3.484	0.297
Smoking history (nonsmoker vs smoker)	1.481	0.699 to 3.137	0.305
Disease site	1.025	0.763 to 1.377	0.872
TNM stage	1.715	1.179 to 2.492	0.005
Lymph node metastasis (pN0 vs pN1 to pN2)	2.596	1.238 to 5.445	0.012
Pathological differentiation	2.294	1.448 to 3.634	0.001
PTHLH expression (low vs high)	0.022	0.003 to 0.159	<0.001
**Multivariate analysis**			
Overall survival			
PTHLH expression (low vs high)	0.024	0.003 to 0.180	<0.001
Pathological differentiation	2.156	1.268 to 3.666	0.005
Lymph node metastasis (pN0 vs pN1 to pN2)	1.100	0.423 to 2.861	0.845
TNM stage	1.193	0.730 to 1.949	0.482

*Abbreviations*: *HR* hazard ration, *CI* confidence interval, *N* lymphnode, *TNM* tumor-lymph node-metastasis classification.

### Positive of HPV16/18 DNA and p16 expression in HNSCCs

For ISH analysis, positive for HPV16/18 was defined if the tumor nuclei were stained. Of the 93 HNSCC samples, 2 cases were positive for the HPV16/18 type and p16 overexpression which detected in both the tumor nuclei and the cytoplasm (Figure 4C). These two patients were both female and with tumors located in base of tongue, the age of 2 patients with HPV-positive HNSCC were respectively 41 and 42 years old. Both of them had no history of smoking and alcohol, and the follow-up time was both 84 months. These data were consistent with the results that HPV16/18 was an important etiologic factor especially for oropharyngeal SCC, and HPV-related HNSCC was more frequent in younger patients with no exposure of tobacco and alcohol [30,31].

## Discussion

PTHLH, which is located on 12p12.1-11.2, is produced by a variety of normal tissues and many normal cells, such as endometrial stromal cells [32]. This gene is responsive to changes based on the extracellular calcium concentration, and it plays important roles at local sites, such as bone, teeth and mammary glands [33]. It has been reported that the expression of PTHrP was regulated by CaR signaling in normal epithelial and cancer cells. According to that report, PTHrP played an important role in the regulation of calcium metabolism in fish and pituitary-related tumors as well as cell lines [34]. The expression of PTHrP was first identified in a lung cancer cell line in 1987 [35]. It was then investigated and analyzed in other cancers and cancer-relative cell lines, such as breast cancer, prostate cancer, ovarian cancer and so on [36]. For example, PTHLH was found to be up-regulated in breast cancers by genome-wide association studies (GWAS) [37]. Lindemann reported that PTHrP expression was regulated by transforming growth factor β through Smad/ets synergism in MDA-MB-231 breast cancer cells [38], but in MCF-7 breast cancer cells, it was shown that PTHrP appeared to function as a osteolytic factor associated with breast cancer metastases via the Smad and MAPK (Mitogen-activated Protein Kinase) signaling pathways [39]. In breast cancers, PTHrP produced by cancer epithelial cells in an autocrine or paracrine manner could induce the local osteolysis that led to the hypercalcemia of malignancy and bone metastases [40]. Bryden reported that PTHrP and its receptor was highly expressed in primary prostate cancer and played a role in the pathogenesis of bone metastases [41]. In 2005, Kornberg et al. found that PTHLH was up-regulated by interrogation of Affymetrix Gene Chip Arrays and further confirmed by real-time PCR and IHC in OSCC [42]. Yamada et al. found that PTHrP was abundantly detected in most of quiescent oral cancer cells and significantly up-regulated by EGF stimulation via ERK and p38 MAPK, and found that PTHrP contributed to the malignancy of oral cancers, for example cell proliferation, migration and invasiveness [43]. In addition, Trivedi et al. found that PTHrP expression was associated with percentage of tumor cells in metastatic lymph nodes from patients with HNSCC [44]. In another work, it was shown that PTHrP affected the growth and vascularity of malignant tumors [45].

In our present study, we found that the PTHLH mRNA and PTHrP protein levels were up-regulated in nearly 88.9% of OSCCs and in all 9 OSCC cell lines compared with the apparently normal surgical margins and normal epithelial cells, respectively. This finding was consistent with other reports showing that PTHLH/PTHrP was overexpressed in many types of cancer, such as breast cancer, esophageal cancer and prostate cancer. Furthermore, this result also verified our previous result showing that PTHLH was up-regulated in OSCC specimens, which indicated that PTHLH/PTHrP might play a role in the pathogenesis of OSCC. Currently, it is still unclear how PTHLH/PTHrP influences cancerous cells, in particular, the biological function of PTHLH/PTHrP in OSCC remains unclear. In this study, we found that down-regulation of PTHLH/PTHrP by RNA interference could inhibit cell growth and colony formation *in vitro*. Both cell growth curves and colony formation efficiency analyses showed that PTHLH/PTHrP affected cell proliferation of OSCC cell lines. More importantly, we found that down-regulation of PTHLH/PTHrP could block the cell cycle at G1 phase and that it could lead to changes in cell cycle-related proteins, including down-regulation of CCND1 and CDK4 and up-regulation of p21, as shown by western blotting and immunocytochemistry. These results suggested that PTHLH/PTHrP could inhibit cell proliferation and reduce colony formation of OSCC cell lines by blocking the cell cycle. These findings were in accordance with the reports showing that PTHLH/PTHrP might act as a growth factor in many cancers and might affect the growth of cancer cells [38,43]. Beyond that, in this study, we found that PTHLH gene may be directly or indirectly involved in the regulation of cell cycle signal pathway. According to these results, we assume that the overexpression of PTHLH/PTHrP promotes the cell cycle of cancer cells, resulting in accelerated progression of OSCC.

To evaluate the clinical significance of the up-regulation of PTHrP in OSCC specimens, we found that high PTHrP expression was positively correlated with pathological differentiation. The expression of the PTHrP protein gradually increased with the degree of pathological differentiation, which was consistent with a report on primary prostate cancer that suggested that PTHrP expression was associated with pathological differentiation [46]. This result implied that patients with high PTHrP expression often had a poor prognosis. In addition, overall survival analysis revealed that high PTHrP expression was associated with poor prognosis, and patients with high PTHrP expression had a significantly low survival. But in 36 paired clinical samples, we did not observed the correlation between PTHLH mRNA expression and pathological differentiation or other clinical features because of the limitation of sample size and some samples had incomplete clinical information. The results of the univariate and multivariate Cox proportional hazards analyses both illustrated that PTHrP expression could be used as an independent indicator of prognosis for patients with HNSCC, which was in accordance with reports showing that high PTHrP expression was an adverse prognostic indicator of gastroesophageal carcinoma [47] and predicted poor survival for patients with early breast cancer [48]. However, these results were in contrast to the 10-year prospective study of Henderson M, who found that women with PTHrP-positive primary breast cancers had a more favorable prognosis and fewer metastases to bone and other sites than patients with PTHrP-negative cancers [49]. This inconsistency might be caused by different tumor types and/or the sample size. A further study involving a much larger patient sample size is required to evaluate this possible association.

Recently, it had been found that HPV was a major etiologic factor for head and neck squamous cell carcinoma especially for oropharyngeal squamous cell carcinoma (OPSCC), and patients with positive HPV show significant better overall survival than those with negative HPV in those cancers [30,50,51]. High-risk HPV16 and HPV18 were the most common types in some cancers, and p16 expression was the surrogate marker for HPV positive in such tumors [52,53]. The contribution of HPV for OPSCC was disagreement in different countries. For example, about 60% of the patients were positive for HPV DNA in the United States [31]. But for Asian populations, the proportion of HPV-related patients was lower compared with Western countries. For example, Kawakami et al. reported that HPV DNA was detected by PCR analysis in 38% (40/104) Japanese patients with OPSCC, and p16 detected by IHC was associated with HPV DNA positivity [51]. Saito et al. reported that the prevalence of Japanese patients with OPSCC positive for HPV was 31% [54]. This inconsistency might be caused by different life habits such as the exposure of tobacco, alcohol and HPVs. In this study, the combined methods of ISH for HPV16/18 and IHC for p16 were used to improve the sensitivity and reliability. Of the 93 HNSCCs, 2 samples were positive for HPV16/18 by ISH as well as p16 expression detected by IHC. These results were in accordance with those of Xu et al. who reported that only 3.5% HPV-related HNSCCs in Chinese patients were detected using the HPV genotyping test [55]. In addition, Li et al. reported that none of 16 tonsil cancer samples were positive for HPV DNA in Chinese patients [56]. The lower proportion of HPV-related patients in our study compared with the result of Kawakami was attributed to the different site of tumors. In Kawakami’s study, the cases were all OPSCC including the tumors of tonsil, posterior wall, lateral wall, base of tongue, anterior palatine arch and so on. In our study, the most majority of cases were tongue squamous cell carcinoma and few tumors originated in tonsil, posterior wall, lateral wall, anterior palatine arch. In fact, our results that HPV16/18 was positive in 2 of 7 tumors originated in base of tongue were consistent with the results of Kawakami who found 4 tumor specimens were positive for HPV in 13 tumors located in base of tongue [51]. Thus it can be seen that HPV is not the important risk factor for Chinese patients with HNSCC. In this study, Kaplan-Meier analysis showed that the HNSCC patients with low PTHrP expression still had better overall survival than those of the patients with high PTHrP expression after adjusting the HPV infectious factor.

## Conclusions

In this study, we found that PTHLH/PTHrP was overexpressed in 88.9% of OSCC tissues and in all 9 OSCC cell lines. Down-regulation of PTHLH/PTHrP could block the cell cycle at G1 phase, inhibit cell proliferation and reduce colony formation of OSCC cell lines *in vitro*. In addition, we found that PTHrP expression was associated with pathologic differentiation and prognosis; to some extent, it could function as an independent biomarker of prognosis for patients with HNSCC. In the future, PTHLH/PTHrP is expected to be a novel molecular target for the development of novel therapeutic strategies.

## Abbreviations

ANOVA: Analysis of variance
CCND1: Cyclin D1
DMEM: Dulbecco’s Modified Eagle medium
DAB: Diaminobenzidine
EGF: Epithelial growth factor
FBS: Fetal bovine serum
GWAS: Genome-wide association studies
HPV: Human papilloma virus
HNC: Head and neck cancer
ISH: In situ hybridization
KSF: Keratinocyte serum-free medium
MAPK: Mitogen-activated Protein Kinase
MMP: Matrix metalloproteinase
OD: Optical densities
OPSCC: Oropharyngeal squamous cell carcinoma
OSCC: Oral squamous cell carcinoma
PBS: Phosphate-buffered saline
PTH: Parathyroid hormone
PTHLH: Parathyroid hormone-like hormone
PTHrP: Parathyroid hormone-related peptide
PVDF: Polyvinylidene fluoride
PCR: Polymerase chain reaction analysis
rEGF: Recombinant epidermal growth factor
SDS: Sodium dodecyl sulfate
SDS-PAGE: Sodium dodecyl sulfate-polyacrylamide gel electrophoresis
siRNA: Small interfering RNA
